# Strontium ranelate effect on bone mineral density is modified by previous bisphosphonate treatment

**DOI:** 10.1186/2193-1801-3-676

**Published:** 2014-11-18

**Authors:** Lucas R Brun, Ana M Galich, Eduardo Vega, Helena Salerni, Laura Maffei, Valeria Premrou, Pablo R Costanzo, Marcelo A Sarli, Paula Rey, María S Larroudé, María S Moggia, María L Brance, Ariel Sánchez, Grupo Argentino de Estudio del Ranelato de Estroncio (GAERE)

**Affiliations:** Laboratorio de Biología Ósea, Facultad de Ciencias Médicas, Universidad Nacional de Rosario, Rosario, Argentina; Servicio de Endocrinología del Hospital Italiano de Buenos Aires, Buenos Aires, Argentina; CESAN, Buenos Aires. Instituto de la Mujer, Campana, Argentina; Consultorios de Investigación Clínica Endocrinológica y del Metabolismo Óseo (CICEMO), Buenos Aires, Argentina; Consultorios Asociados de Endocrinología Dra. Laura Maffei, Buenos Aires, Argentina; Instituto de Investigaciones Metabólicas Dr. Zanchetta, Buenos Aires, Argentina; Hospital César Milstein, Buenos Aires, Argentina; Centro Tiempo, Buenos Aires, Argentina; Centro de Reumatología, Rosario, Argentina; Centro de Endocrinología, Rosario, Argentina

**Keywords:** Strontium ranelate, Bone mineral density, Bisphosphonate-naïve

## Abstract

The aim of this study was to evaluate the effect of strontium ranelate (SrR) on bone mineral density (BMD) and bone turnover markers after 1 year of treatment. Additionally, the effect of SrR in bisphosphonate-naïve patients (BP-naïve) compared to patients previously treated with bisphosphonates (BP-prior) was analyzed. This retrospective study included 482 postmenopausal women treated with SrR (2 g/day) for 1 year in ten Argentine centers; 41 patients were excluded due to insufficient data, while 441 were included. Participants were divided according to previous bisphosphonate treatment in two groups: BP-naïve (n = 87) and BP-prior (n = 350). Data are expressed as mean ± SEM. After 1 year of treatment with SrR the bone formation markers total alkaline phosphatase and osteocalcin were increased (p < 0.0001), while the bone resorption marker s-CTX was decreased (p = 0.0579). Also increases in BMD at the lumbar spine (LS, 3.73%), femoral neck (FN, 2.00%) and total hip (TH, 1.54%) [p < 0.0001] were observed. These increments were significant (p < 0.0001) both among BP-naïve and BP-prior patients. Interestingly, the change in BMD after 1 year of SrR treatment was higher in BP-naïve patients: LS: BP-naïve = 4.58 ± 0.62%; BP-prior = 3.45 ± 0.28% (p = 0.078). FN: BP-naïve = 2.79 ± 0.56%; BP-prior = 2.13 ± 0.29% (p = 0.161). TH: BP-naïve = 3.01 ± 0.55%; BP-prior = 1.22 ± 0.27% (p = 0.0006). SrR treatment increased BMD and bone formation markers and decreased a bone resorption marker in the whole group, with better response in BP-naïve patients.

## Introduction

Osteoporosis is a chronic condition characterized by decreased bone mass and deterioration of bone microarchitecture; it compromises bone strength thus predisposing to fragility fractures. Current available and worldwide approved treatments for osteoporosis are antiresorptive medications, which include bisphosphonates (BP), selective estrogen-receptor modulators, calcitonin and denosumab, and bone-forming agents, such as teriparatide (PTH_1–34_) (Schurman et al. 2013). Strontium ranelate (SrR) is widely used for the treatment of postmenopausal osteoporosis (Galich 2011). Its mechanism of action is dual, since on one hand it induces bone formation (anabolic effect) while on the other hand it reduces the rate of bone resorption (anticatabolic effect) (Bonnelye et al. 2008). Its efficacy has been demonstrated in relatively young and elderly (80 and more years old) women, in patients with high fracture or refracture risk, in smokers, and in men (Meunier et al. 2004, 2009; Reginster et al. 2005; Seeman et al. 2006; Kaufman et al. 2013). It has been shown to be effective in reducing the incidence of vertebral and non-vertebral fractures (Meunier et al. 2004; Reginster et al. 2005).

SrR decreases osteoclast differentiation and activity *in vitro*, as demonstrated by decrements of bone resorption markers but not bone formation markers in ovariectomized rats (Marie et al. 1993). Additionally it induces the disruption of osteoclast cytoskeleton and decreases osteoclast resorbing activity (Takahashi et al. 2003). SrR increases the production of osteoprotegerin (OPG) and reduces the expression of receptor activator of nuclear factor κB ligand (RANKL) in osteoblasts (Atkins et al. 2009; Brennan et al. 2009). It has been reported that OPG is increased in postmenopausal women treated with SrR as early as 3 months after initiation of treatment (Reginster et al. 2012). Additionally, SrR increases the replication of preosteoblasts, increases bone matrix synthesis by preosteoblasts and osteoblasts (Canalis et al. 1996) and increases mineralization (Choudhary et al. 2007). SrR induces osteoblastogenesis by stimulation of both canonical and noncanonical Wnt signaling pathways (Fromigué et al. 2010). Accumulation of advanced glycation endproducts (AGEs) in bone tissue occurs in ageing and in diabetes mellitus. It has been demonstrated that SrR can prevent the deleterious in vitro actions of AGEs on osteoblastic cells in culture by mechanisms that involve calcium channel, MAPK and β-catenin activation (Fernández et al. 2013).

Significantly higher mineral apposition rate in cancellous bone was observed by histomorphometry. Using μCT of bone biopsies collected from humans receiving long-term treatment with SrR over 5 years, increase in cortical thickness and trabecular number has been demonstrated, with no change in cortical porosity (Arlot et al. 2008). Also, greater effects on distal tibia cortical thickness and trabecular volumetric density were observed with SrR versus alendronate over 2 years using HR-pQCT (Rizzoli et al. 2010).

Many osteoporotic women previously treated with BP are prescribed SrR. Those patients who have adverse effects of BP, or who maintain high fracture risk or have poor treatment response to BP are of particular interest. Middleton et al. have demonstrated that after treatment with SrR the bisphosphonate-naïve group (BP-naïve) had significantly greater bone mineral density (BMD) increments in spine, hip and heel (Middleton et al. 2010, 2012).

The aim of this study was to evaluate the effect of SrR on BMD and bone turnover markers after 1 year of treatment in clinical practice conditions in specialized centers from Argentina. Additionally, the effect of SrR in BP-naïve patients compared to patients previously treated with bisphosphonates (BP-prior) was analyzed.

## Patients and methods

This retrospective study analyzed records from 482 postmenopausal women treated with SrR (2 g/day) for 1 year in ten Argentine centers. All women had either a T-score of less than -2.5 at the hip or spine or a T-score of less than -2.0 and other risk factors for fracture. All patients simultaneously received calcium (1000 mg/day) and vitamin D (800 U/day). Women were excluded if they had medical conditions or took medications associated with bone disease. Participants were also analyzed considering the previous use of BP; they were divided in BP-naïve (n = 87) and BP-prior (n = 350) patients; 4 women were not included in this analysis because they used other drugs besides BP.

Antrophometric parametes were considered: weight (kg), height (m). Body mass index (BMI) was calculated according to the formula: weight/height^2^ (kg/m^2^).

BMD (g/cm^2^) was measured by dual-energy X-ray absorptiometry (DXA) GE Lunar Prodigy (GE Lunar, Madison, WI, USA) in lumbar spine (L2-L4), femoral neck and total hip. The coefficient of variation was less than 3% in all centers where densitometries were performed.

Plasma calcium levels (mg/dl), plasma phosphate levels (mg/dl) and total alkaline phosphatase (tAP, UI/L) were spectrophotometrically measured. Serum parathyroid hormone (iPTH, pg/ml) was measured by chemoluminiscent assay (iPTH Siemens Medical Solutions Diagnostics). Total serum 25-hydroxyvitamin D levels [25(OH)D, ng/ml] and serum carboxy-terminal crosslinking telopeptide of type I collagen (s-CTX, ng/l) were measured by electrochemoluminiscent assay (Elecsys® Total Vitamin D Roche, and Elecsys® ϐ-CrossLaps Roche Diagnostics, respectively). Serum osteocalcin (BGP, ng/ml) was determined by electrochemoluminiscent assay (Roche Diagnostics). All measurements were not made in the same place and by the same person, although the same methods were used.

### Data analysis

Data are expressed as mean ± SEM and were analyzed with Mann–Whitney test or Wilcoxon signed rank test as appropriate. Kolmogorov-Smirnov test for normality was used to assess the distribution of the data. Differences were considered significant if p < 0.05. Statistical analyses were performed with GraphPad Prism 2.0 (GraphPad, San Diego, USA).

## Results

### Subjects and baseline clinical characteristics

Medical records from 482 postmenopausal were analyzed; 41 patients were excluded due to insufficient data, while 441 were included. The main characteristics of the study population are shown in Table 1.

**Table 1:** **Baseline clinical characteristics of all patients (n = 441)**

	Basal
Age (years)	67.20 ± 0.50
Body mass index (kg/m^2^)	24.55 ± 0.18
Serum calcium (mg/dl)	9.36 ± 0.02
Urinary calcium (mg/24 h)	171.30 ± 5.20
Serum phosphate (mg/dl)	3.97 ± 0.03
25(OH) vitamin D (ng/ml)	32.04 ± 1.00
iPTH (pg/ml)	51.00 ± 3.11
tAP (IU/L)	59.70 ± 1.36
BGP (ng/ml)	17.02 ± 0.98
s-CTX (ng/l)	331.10 ± 16.03
Lumbar spine BMD [g/cm^2^; T-score]	0.859 ± 0.005; -2.75 ± 0.04
Femoral neck BMD [g/cm^2^; T-score]	0.718 ± 0.004; -2.29 ± 0.04
Total hip BMD [g/cm^2^; T-score]	0.747 ± 0.005; -2.17 ± 0.05

### Change in bone markers with SrR

**Figure 1:**
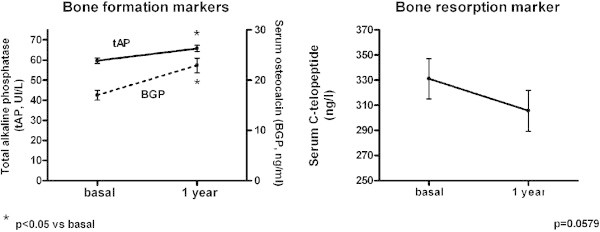
Increase of bone formation markers and decrease of a bone resorption marker after 1 year of treatment with SrR (*p < 0.05 vs basal).

After 1 year of treatment with SrR, the bone formation markers tAP (65.76 ± 1.57 UI/L) and BGP (22.93 ± 1.46 ng/ml) were significantly increased (Wilcoxon signed rank test, p < 0.0001), with a mean increment of 10.15% and 34.72%, respectively. Meanwhile, the bone resorption marker s-CTX (305.60 ± 16.31 ng/l) decreased by 7.7%, although this was not significant (Wilcoxon signed rank test, p = 0.0579; Figure 1).

### Change in BMD with SrR

**Figure 2:**
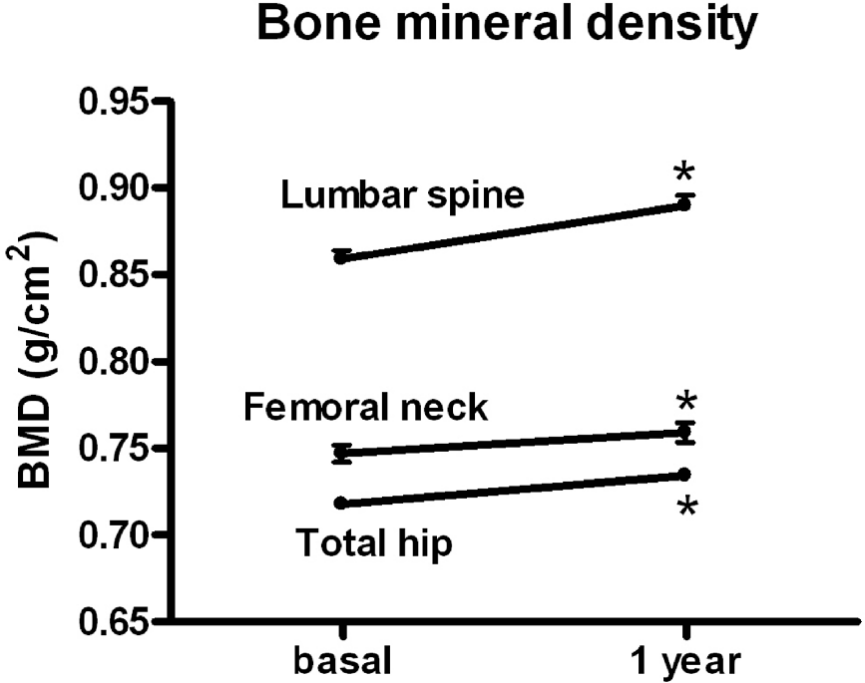
BMD increase at all sites after 1 year of treatment with SrR (*p < 0.05 vs basal).

After 1 year of treatment with SrR an increased BMD at the lumbar spine (LS), femoral neck (FN) and total hip (TH) was observed (Wilcoxon signed rank test, p < 0.0001). The percent change in BMD was: LS = 3.73 ± 0.26%, FN = 2.00 ± 0.24% and TH = 1.54 ± 0.24% (Figure 2).

### Bisphosphonate-naïve vs prior bisphosphonate patients: main characteristics

The patients were also analyzed considering the previous use of BP; they were divided in BP-naïve (n = 87) and BP-prior (n = 350) patients; 4 patients were not included in this analysis because they used other drugs besides BP. The duration of previous BP treatment was 5.35 ± 0.24 years. The age was higher in BP-prior group: BP-naïve = 60.45 ± 1.12 years; BP-prior = 69.00 ± 0.56 (Mann Whitney test, p < 0.0001). There were no significant differences in BMI, years of menopause, serum calcium, urinary calcium, serum phosphate, 25(OH)D and iPTH between groups (data not shown).

### Bisphosphonate-naïve vs prior bisphosphonate patients: bone markers

As expected, basal BGP (but not tAP) was lower (p = 0.05) in the BP-prior group due to previous antiresorptive treatment. After 1 year of treatment with SrR both BPG and tAP, as bone formation markers, were increased, particularly in the BP-prior group (BPG: BP-naïve: 39.5% (p = 0.08); BP-prior: 38.4% (p = 0.002); tAP = BP-naïve: 4.4% (p = 0.46) and BP-prior: 10.7% (p = 0.005). Similarly, basal s-CTX was lower (p = 0.005) in the BP-prior group. After 1 year of treatment with SrR this bone resorption marker was decreased; although this change was not significant: BP-naïve: -10.8% (p = 0.40) and BP-prior: -12.72% (p = 0.11) (Table 2).

**Table 2:** **Changes in bone markers after 1 year of SrR treatment**

	BP-naïve	***p***	BP-prior	***p***
tAP	↑ 4.4%	0.46	↑ 10.7%	0.005
BGP	↑ 39.5%	0.08	↑ 38.4%	0.002
s-CTX	↓ 10.8%	0.40	↓ 12.7%	0.11

### Bisphosphonate-naïve vs prior bisphosphonate patients: bone mineral density

**Figure 3:**
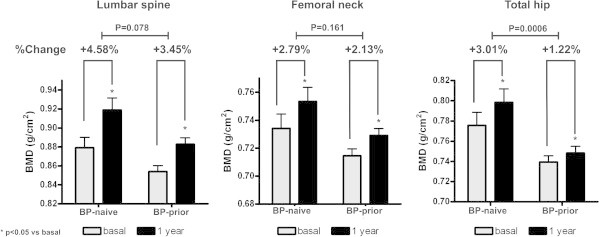
A better response in BP-naïve patients was observed after 1 year of treatment with SrR (*p < 0.05 vs basal).

The increments in BMD in the whole group at the LS, FN and TH was also found both among BP-naïve and BP-prior patients (Wilcoxon signed rank test, p < 0.0001; Figure 3).

Interestingly, the change in BMD after 1 year of treatment with SrR was higher in BP-naïve patients: LS: BP-naïve = 4.58 ± 0.62%; BP-prior = 3.45 ± 0.28% (Mann–Whitney test, p = 0.078). FN: BP-naïve = 2.79 ± 0.56%; BP-prior = 2.13 ± 0.29% (Mann–Whitney test, p = 0.161). TH: BP-naïve = 3.01 ± 0.55%; BP-prior = 1.22 ± 0.27% (Mann–Whitney test, p = 0.0006; Figure 3).

## Discussion

The study evaluated the effect of SrR on BMD and bone turnover markers after 1 year of treatment in clinical practice conditions. A good response in BMD was observed after 1 year of SrR treatment at all studied regions: LS = 3.73%, FN = 2.00% and TH = 1.54%. Coincident with BMD changes, an increase of bone formation markers and a decrease of the selected bone resorption marker were found. Borderline changes in s-CTX (p = 0.0579) can be attributed to the wide dispersion of data.

When BMD was analyzed considering the previous use of BP, a better response in BP-naïve patients was observed, in accordance with previous papers. In agreement with Middleton et al’s report (Middleton et al. 2010) our study found similar changes at the LS (4.58 vs. 5.6%) and TH (3.01 vs. 3.4%) in the BP-naïve group, and also at the LS (1.22 vs. 0.8%) in the BP-prior group. Although Middleton et al. did not find differences in TH BMD values after 1 year of treatment with SrR, a significant difference was observed in our study (p = 0.0006). Besides, our study did not show a significant increase at the LS in BP-naïve patients after 1 year of treatment, but a borderline *p* value was found (p = 0.078). Middleton et al. (Middleton et al. 2012) published data after 2 years of treatment, where BMD increased significantly from baseline in both groups, BP-naïve (LS = 8.9%; TH = 6.0%) and BP-prior (LS = 4.0%; TH = 2.5%). When they compared BP-naïve vs. BP-prior they found higher increments among BP-naïve patients at 6 months of SrR treatment, persisting after 24 months (Middleton et al. 2012). This suggests that the differences found between our study and Middleton et al’s (Middleton et al. 2010) could be the consequence of differences in size and variability, and particularly of longer treatment time with SrR, because after 2 years the differences become more evident.

There were differences in age between BP-naïve and BP-prior women; however, it is unlikely that this could have influenced the results: in the SOTI and TROPOS studies the gain in BMD at the spine and the hip was of the same magnitude among women 80 years or older than in younger osteoporotic women (Seeman et al. 2006).

Similarly, after 1 year of treatment, teriparatide induced a mean gain in lumbar spine BMD that was greater in the BP-naïve group (8.4%) than in patients pretreated with antiresorptives with no evidence of inadequate treatment response (7.1%), and in patients pretreated but showing an inadequate response to antiresorptives (6.2%). Total hip BMD increased from baseline in the BP-naïve (1.8%) but no changes was observed in the groups BP-prior (-0.3% and 0.4%) (Minne et al. 2008).

The use of BP reduces bone turnover leading to reduced new bone formation (Li et al. 2010) which could reduce strontium uptake. However, markers of bone formation were increased particularly in the BP-prior group (Table 1). This is consistent with previous studies (Middleton et al. 2010, 2012) and could indicate important changes in bone turnover due to its previous status under antiresorptive treatment. Suppressed bone turnover could be the cause of a blunted response to SrR treatment, but it seems to be reversed after 6 months according to procollagen type 1 amino terminal propeptide (P1NP) measurements (Middleton et al. 2012). However, the BP-prior group could not reach the same values as the BP-naïve group at least after 2 years of treatment with SrR (Middleton et al. 2012).

Paired iliac crest biopsies from 15 patients previously treated with BP suggest that SrR generates new bone (Busse et al. 2010). This study found an increase in osteoid surface and strontium content after 6 months of treatment. After 12 months of SrR administration, there was a significant increase in bone volume and trabecular thickness, increased number of osteoblasts and osteoid surface and volume.

There are limitations to this study. This was not a prospective study, and BMD and bone markers were recorded after 1 year without intermediate measurements. The wide dispersion of some parameters could be due to the fact that measurements were not made in the same place and by the same person, although the same methods were used. Also, the number of the BP-naïve women is not similar to that of BP-prior women. This discrepancy could influence the significance of results. Finally, it should be considered that most of the women in the BP-prior group were switched to SrR because of poor clinical response to BP. There could be an undeterminate difference between groups in the response to treatment.

In conclusion, SrR treatment increased BMD and bone formation markers and decreased bone resorption marker in the whole group with better response in BP-naïve patients. Since there are no head-to-head studies between BP and SrR comparing fracture risk reduction, it is important to evaluate individual patients taking into consideration expert guidelines either before choosing the first treatment or before changing to a new one.
